# Increased serum HO-1 in hemophagocytic syndrome and adult-onset Still's disease: use in the differential diagnosis of hyperferritinemia

**DOI:** 10.1186/ar1721

**Published:** 2005-03-21

**Authors:** Yohei Kirino, Mitsuhiro Takeno, Mika Iwasaki, Atsuhisa Ueda, Shigeru Ohno, Akira Shirai, Heiwa Kanamori, Katsuaki Tanaka, Yoshiaki Ishigatsubo

**Affiliations:** 1 Department of Internal Medicine and Clinical Immunology, Yokohama City University Graduate School of Medicine, Yokohama, Japan; 2 Yokohama City University Medical Center, Department of Gastroenterological Center, Yokohama, Japan

## Abstract

Heme oxygenase-1 (HO-1), an inducible heme-degrading enzyme, is expressed by macrophages and endothelial cells in response to various stresses. Because ferritin synthesis is stimulated by Fe^2+^, which is a product of heme degradation, we examined the relation between HO-1 and ferritin levels in the serum of patients with hemophagocytic syndrome (HPS), adult-onset Still's disease (ASD), and other diseases that may cause hyperferritinemia. Seven patients with HPS, 10 with ASD, 73 with other rheumatic diseases, 20 with liver diseases, 10 recipients of repeated blood transfusion because of hematological disorders, and 22 healthy volunteers were enrolled. Serum HO-1 and ferritin levels were determined by ELISA. Expression of HO-1 mRNA and protein by peripheral blood mononuclear cells (PBMCs) was determined by real-time PCR and immunocytochemical techniques, respectively. Serum levels of HO-1 were significantly higher in patients with active HPS and ASD than in the other groups (*P *< 0.01). HO-1 levels were not elevated in patients with other causes of hyperferritinemia but were moderately elevated in patients with dermatomyositis/polymyositis. Among patients with HPS and ASD, serum HO-1 levels correlated closely with serum ferritin levels, and the levels of both returned to normal after therapy had induced remission. Increased expression of HO-1 mRNA was confirmed in PBMCs from some patients with HPS and ASD. Hyperferritinemia correlated closely with increased serum HO-1 in patients with HPS and ASD but not other conditions, indicating that measurement of serum HO-1 and ferritin levels would be useful in the differential diagnosis of hyperferritinemia and perhaps also in monitoring disease activity in HPS and ASD.

## Introduction

Heme oxygenase (HO) is an enzyme that catalyzes the conversion of heme into CO, Fe^2+^, and biliverdin [1,2]. HO-1, an inducible form of HO, is a 32-kD heat shock protein expressed in response to various noxious stimuli including heavy metals, hyperoxia, hypoxia, endotoxin, hydrogen peroxide, and inflammatory cytokines [1,2]. Evidence suggests that increased expression of HO-1 can benefit the host in a variety of pathological conditions [1-5]. In this context, our research team has found that HO-1 gene therapy is useful for lipopolysaccharide-induced lung injury [6], influenza viral pneumonia [7], bleomycin-induced pulmonary fibrosis [8], and chronic respiratory infection with *Pseudomonas aeruginosa *in mice [9]. We also found that chemically induced HO-1 was of benefit in lupus nephritis [10]. On the other hand, a deficiency in HO-1 expression is associated with severe chronic inflammation, as shown in studies of HO-1 knockout mice (mice in which the gene for HO-1 had been inactivated) and a patient with HO-1 deficiency [11-13]. This observation is consistent with HO-1 having a physiological effect in protecting against inflammation.

Products of heme degradation mediate the protective effects of HO-1. CO suppresses apoptosis, macrophage activation, and the synthesis of proinflammatory cytokines, nitrite oxide, and prostaglandins [1,2,14]. Biliverdin is converted into bilirubin, an antioxidant [1,2,15-18]. Fe^2+^, which itself has toxic effects by inducing the formation of free radicals, stimulates the production of ferritin [19]. Ferritin acts as an antioxidant and detoxifies Fe^2+ ^[19]. Thus, the heme degradation products and the metabolic derivatives generated by HO-1 suppress toxic events in cells.

Regulation of HO-1 is of particular interest in the inflammation associated with hyperferritinemia, as is the case in hemophagocytic syndrome (HPS) and adult-onset Still's disease (ASD), because HO-1 can be involved in increased ferritin in these conditions [1,2]. HPS is a serious, life-threatening condition, which is characterized by cytopenia due to hemophagocytosis [20-22]. The disease is subdivided into two categories, familial lymphohistiocytosis and secondary HPS, the latter of which is associated with rheumatic diseases such as systemic-onset juvenile idiopathic arthritis, viral infection, and certain malignancies [20].

Like children with Still's disease, patients with ASD present with high fever, arthralgia, typical skin rash, hepatosplenomegaly, and leukocytosis [20,21]. HPS and ASD share several clinical features, including high fever, hepatosplenomegaly, lymphadenopathy, liver injury, and coagulopathy [20,21]. The observation that severe ASD is sometimes complicated by HPS is consistent with the suggestion that a common pathophysiology may link these two diseases [20,21,23].

Recent studies have shown that dysfunction of natural killer (NK) cells due to mutations of the genes for perforin and Munc 13-4 leads to familial lymphohistiocytosis, whereas it has been suggested that decreased NK cell activity and abnormal levels of perforin are involved in the macrophage activation syndrome of systemic-onset juvenile rheumatoid arthritis [20]. Dysfunction of NK and cytotoxic cells may lead to inadequate control of cellular immune responses, resulting in systemic macrophage activation, which is implicated in the development of both diseases of HPS and ASD. Subsequently, excessive production of proinflammatory cytokines and active infiltration of macrophages into vital organs have been observed [20,21]. Increased serum ferritin is characteristic of, but not specific for, both diseases, because it is also elevated in various other conditions [23,24]. For example, patients with hyperferritinemia who have rheumatic or liver disease or who receive frequent transfusions because of hematological diseases often develop cytopenia and high fever resembling these signs in HPS.

Lack of specific disease markers often delays diagnosis of HPS and ASD, with potentially lethal consequences [21]. The present study shows that serum HO-1 levels are significantly increased in patients with active HPS and ASD but not in patients with hyperferritinemia due to other causes. Moreover, there is a close correlation between serum HO-1 levels and the disease activity in HPS and ASD.

## Materials and methods

### Patients

All patients enrolled in this study were being treated at the Yokohama City University Hospital, the Yokohama City University Medical Center Hospital, or the National Hospital Organization Yokohama Medical Center (Table 1). Seven patients with secondary HPS met the diagnostic guideline for hemophagocytic lymphohistiocytosis [22,25], except as regards hypertriglyceridemia and hypofibrinogenemia, neither of which is generally applicable to secondary HPS in adults. In these seven patients, the underlying diseases were systemic lupus erythematosus (SLE) in two; hematological malignancy, including non-Hodgkin's lymphoma, multiple myeloma, and acute myeloid leukemia, in three; and ASD and viral infection in the others. The patients having more than two lineages of cytopenia, liver dysfunction, fever above 39°C, and hyperferritinemia were categorized as having active disease. Remission of the diseases was defined as disappearance of these findings after therapy. Ten patients with ASD met the criteria of Cush [26] and Yamaguchi [27] and their colleagues. An ASD patient who also met the diagnostic guidelines for hemophagocytic lymphohistiocytosis was classified in the HPS group in this study. Patients with active ASD were those presenting with polyarthritis, typical skin rashes, and fever above 39°C, in addition to hyperferritinemia. When the symptoms and signs had subsided, the patients were considered to be in remission.

We also studied 73 patients with other rheumatic diseases, including 30 with rheumatoid arthritis (RA), 18 with SLE, 9 with dermatomyositis/polymyositis (DM/PM), and 16 with Behçet's disease (BD). The diagnosis of individual diseases was based on the following criteria: for RA, the 1987 American College of Rheumatology (formerly, the American Rheumatism Association) criteria [28]; for SLE, the 1997 updating of the American College of Rheumatology revised criteria for the classification of systemic lupus erythematosus [29]; for polymyositis and dermatomyositis, the diagnostic criteria described by Bohan and Peter [30,31]; and for Behçet's disease, the International Study Group criteria for diagnosis of Behçet's disease [32]. The disease activity was evaluated at the time of blood sampling. All of the RA patients were considered to have active disease, because their disease activity scores (DAS) based on 28 joints and C-reactive protein (CRP) (DAS28-CRP) were more than 3.2 [33]. The mean CRP level at the time of blood sampling was 2.4 ± 2.7 mg/dl. On the basis of the Systemic Lupus Disease Activity Index [34], 12 of the 18 SLE patients had a score above 9 and were regarded as having active disease, while the other 6 were in remission. Two other SLE patients who met the diagnostic guidelines for hemophagocytic lymphohistiocytosis were included in the HPS group [22,25]. All of the DM/PM patients had active diseases, inasmuch as their creatine kinase concentrations were more than twice the normal upper limit and they had muscle weakness and/or active interstitial pneumonia. Six of the 16 BD patients presented active symptoms of uveitis, erythema nodosum, genital ulcers, deep vein thrombus, central nervous system involvement, arterial occlusion, or gastrointestinal lesions in addition to positive CRP, indicating active disease; the other 10 were regarded as having inactive disease.

Twenty patients with liver diseases were enrolled in this study (Table 1). Of the five with acute hepatitis, three had hepatitis B, one had drug-induced hepatitis, and one had Epstein–Barr viral hepatitis. The seven patients with chronic hepatitis included one with hepatitis B and six with hepatitis C. Serum alanine aminotransferase levels were measured as an indicator of liver injury. The means ± standard deviations (IU/l) found for these 20 patients were as follows: acute hepatitis, 770.0 ± 568.6; chronic hepatitis, 61.9 ± 28.9; liver cirrhosis, 59.0 ± 24.0; hepatocellular carcinoma, 27.5 ± 14.8; primary biliary cirrhosis, 98.5 ± 14.1; autoimmune hepatitis, 259; and alcoholic hepatitis, 56.

Ten patients who had received frequent blood transfusions were also included. The underlying hematological diseases were myelodysplastic syndrome in six patients and aplastic anemia in four. Healthy volunteers served as normal controls. All the studies were performed after obtaining written informed consent, which was approved by the local Institutional Review Board.

### ELISA

Serum ferritin and HO-1 levels were measured by an EIA detection system (Tosoh, Tokyo, Japan), and a human HO-1 ELISA kit (Stressgen, Victoria, Canada), respectively. Concentrations of serum tumor necrosis factor (TNF)-α were determined by specific ELISA systems using pairs of capture and biotin-conjugated detecting antibodies, which were purchased from R&D (Minneapolis, MN, USA). Serum IL-18 level was determined using a human IL-18 ELISA kit in accordance with the manufacturer's protocol (MBL, Nagoya, Japan).

### Cell preparation and culture

Peripheral blood mononuclear cells (PBMCs) were isolated by centrifugation over Ficoll-Hypaque (ICN, Aurora, OH, USA). 10^6^cells/ml were cultured with 100 μM hemin (Sigma-Aldrich, Saint Louis, MO, USA) in Hepes modified RPMI 1640 (Sigma-Aldrich) containing 10% fetal calf serum (Equitech-Bio, Kerrville, TX, USA), 2 mM L-glutamine (Sigma-Aldrich), 100 U/ml penicillin, plus 100 μg/ml streptomycin (Sigma-Aldrich) in a 5% CO_2 _in an air incubator at 37°C for 24 hours.

### Real-time PCR

Total RNA was isolated from cells by using TRIzol reagent (Invitrogen, Carlsbad, CA, USA). Reverse transcription was performed using a SuperScript™ reverse transcriptase (Invitrogen). Panels of primers of human HO-1 and glyceraldehyde-3-phosphate dehydrogenase (GAPDH) mRNA were purchased from PE Applied Biosystems (Foster City, CA, USA). Real-time PCR was performed using a TaqMan Universal Master Mix (PE Applied Biosystems), and the data were analyzed by the ABI prism 7700 sequence detection system (PE Applied Biosystems). Briefly, 1/50 amounts of of cDNA derived from 1 μg of total RNA, 200 nmol/l of probe, and 800 nmol/l of primers were incubated in 25 μl at 50°C for 2min and 95°C for 10min, followed by 40 cycles of 95°C for 15s and 60°C for 1 min. The amounts of cDNA obtained from transcriptions of mRNA were semiquantified in comparison with those of serially diluted standard cDNA, which was prepared using a conventional PCR technique. The expression level of HO-1 mRNA in a sample was expressed as arbitrary units, which were determined by the formula 1AU = (HO-1 mRNA/GAPDH mRNA) × 100.

### Immunocytochemistry

Cells expressing HO-1 were determined with anti-HO-1 monoclonal antibody (Stressgen) using a Dako LSAB2 kit (Dako, Glostrup, Denmark).

### Statistical analysis

The Mann–Whitney *U *test, the Wilcoxon signed rank test, and multiple regression analyses were used to test for differences. *P *values less than 0.05 were considered significant. Values are reported as means ± standard deviations.

## Results

### Increased serum HO-1 levels in patients with HPS and ASD

**Figure 1:**
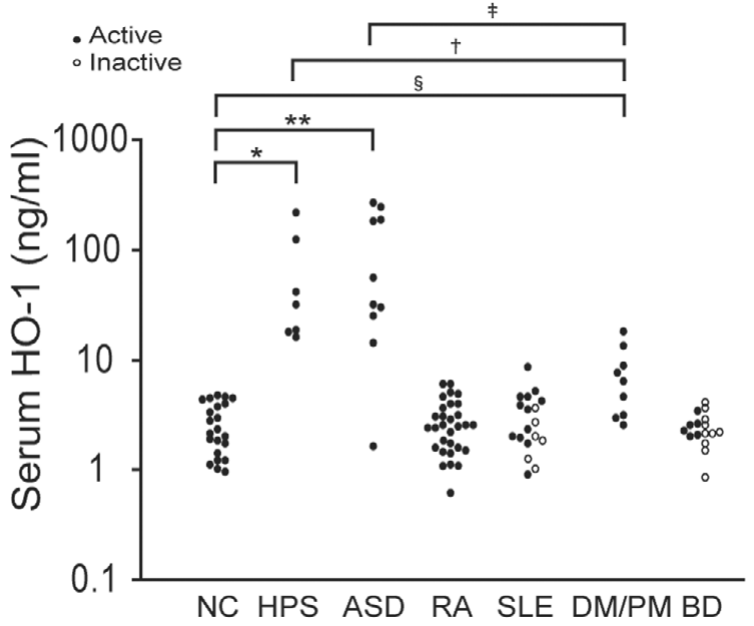
Serum heme oxygenase-1 in patients with hemophagocytic syndrome or adult-onset Still's disease. Also studied were normal controls (NC) and people with other rheumatic diseases including rheumatoid arthritis (RA) (*n *= 30), systemic lupus erythematosus (SLE) (*n *= 18), dermatomyositis/polymyositis (DM/PM) (*n *= 9), and Behçet's disease (BD) (*n *= 16). Filled circles and open circles represent patients with active and inactive disease, respectively. *P < 0.0001, **P = 0.0001, ^§^*P *= 0.001, ^†^*P *= 0.0007, ^‡^*P *= 0.003, as determined by the nonpaired Mann–Whitney *U *test. ASD, adult-onset Still's disease; HO-1, heme oxygenase 1; HPS, hemophagocytic syndrome.

Serum HO-1 levels in patients with inflammatory rheumatic diseases were monitored by ELISA. In the healthy controls, only very low levels of serum HO-1 were detectable (2.6 ± 1.3 ng/ml) (Fig. 1). Age and sex did not influence HO-1 levels. In contrast, HO-1 levels were significantly elevated in patients with active ASD and HPS (Table 1; Fig. 1). HO-1 protein levels exceeded 10 ng/ml in all but one patient with ASD, who was classified as having active disease in the study because of high fever with elevated levels of CRP and ferritin during maintenance therapy with a low dose of prednisolone (PSL). However, the clinical manifestations were less serious and serum ferritin was lower (1201 ng/ml) than in any other patient with active ASD in this study. Although subjects with active DM/PM also had significantly increased serum HO-1 levels (Table 1; *P *= 0.001), these were still significantly lower than in subjects with active HPS or ADS (*P *= 0.0007 and *P *= 0.003, respectively). Serum HO-1 levels were not increased in other rheumatic diseases including RA, SLE, and BD, regardless of disease activity (except for two patients with SLE complicated by HPS) (Table 1). These findings suggest that increased serum HO-1 levels are characteristic of active ASD and HPS.

### Serum HO-1 is a marker of disease severity in HPS and ASD

**Figure 2:**
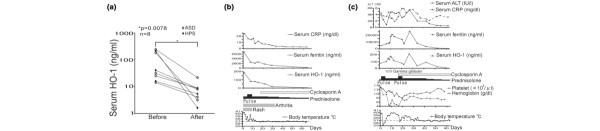
Serum heme oxygenase (HO)-1 and ferritin in hemophagocytic syndrome (HPS) and adult-onset Still's disease (ASD). **(a) **Serum HO-1 levels of HPS patients (open circles) and ASD patients (filled circles) before and after remission. *P = 0.0078, as determined by the Wilcoxon signed-rank test. **(b) **Clinical course of ASD in a 34-year-old man. **(c) **Clinical course in a 45-year-old woman with HPS and systemic lupus erythematosus. 'Pulse' represents intravenous infusion of methylprednisolone at 1,000 mg/day for 3 days. ALT, alanine aminotransferase; CRP, C-reactive protein.

Serum HO-1 levels were monitored before and after remission-inducing therapy that included corticosteroids with or without cyclosporin A in three patients with HPS and five with ASD. Serum HO-1 levels were significantly reduced after successful therapy (Fig. 2a) (*P *= 0.0078).

Serum HO-1 and ferritin were serially monitored in one patient with ASD and one with HPS during the course of disease (Fig. 2b,c). A 34-year-old man admitted with fever, polyarthralgia, sore throat, and salmon-pink rashes was diagnosed with ASD (Fig. 2b). When this patient was admitted, his serum concentrations of both HO-1 and ferritin were extremely elevated (182 ng/ml and 6,855 ng/ml, respectively). Treatment with methylprednisolone (mPSL) pulse therapy (1,000 mg/day for 3 days) followed by oral PSL (60 mg/day) and cyclosporin A (200 mg/day) led to clinical remission. Associated with this response to therapy, serum HO-1 levels gradually decreased to the normal range over 2 months, as did levels of ferritin and CRP. PSL was tapered to 30 mg/day without relapse.

In a 45-year-old woman with SLE admitted with high fever and cytopenia (Fig. 2c), bone marrow aspiration revealed hemophagocytosis, and her serum ferritin level was 4,588 ng/ml, resulting in a diagnosis of HPS complicated with SLE (the Systemic Lupus Disease Activity Index score was 9). On admission, increased serum HO-1 (74.8 ng/ml) was noted. mPSL pulse therapy (1,000 mg/day for 3 days) followed by oral PSL (60 mg/day) and intravenous gamma globulin (17.5 g/day for 5 days) temporarily reduced her fever and CRP levels. Despite these treatments, serum ferritin and HO-1 peaked at 25,070 ng/ml and 214 ng/ml, respectively. A second course of mPSL pulse therapy also failed, but the patient's condition gradually improved after initiation of cyclosporin A (200 mg/day). Serum levels of CRP, ferritin, and HO-1 reached normal levels by two months after admission. PSL was tapered to 30 mg/day without exacerbation. These findings suggest that the serum HO-1 level is closely correlated with disease activity during the clinical course in patients with HPS and ASD.

**Figure 3:**
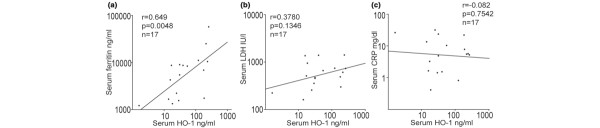
Correlation between serum heme oxygenase (HO)-1 and other serum constituents. **(a) **Correlation between serum HO-1 and ferritin in the patients with hemophagocytic syndrome (HPS) and adult-onset Still's disease (ASD) at the time when the serum ferritin was highest during the study. *P *= 0.0048, as determined by multiple regression analyses. **(b,c) **Correlations between HO-1 and (b) lactate dehydrogenase (LDH), and (c) C-reactive protein (CRP) in the same patients at the same point in the study.

We next examined the relation between the serum HO-1 level and other laboratory parameters in the patients with HPS and ASD. Because serum ferritin was widely accepted as a monitoring marker for the diseases, the data included in the analysis were those found when the ferritin level was highest in individual patients during the whole study. The results indicate that serum HO-1 correlates closely with serum ferritin (*P *= 0.0048, Fig. 3a) but not CRP or lactate dehydrogenase (LDH) levels (Fig. 3b,c), a finding consistent with an association between HO-1 and hyperferritinemia in patients with HPS and ASD. We also measured serum levels of IL-18 and TNF-α, both of which have been shown to be elevated in patients with HPS and ASD [35,36]. However, we did not find any correlation between serum level of HO-1 and those of cytokines (data not shown).

### Increased serum HO-1 level is not always associated with hyperferritinemia

**Figure 4:**
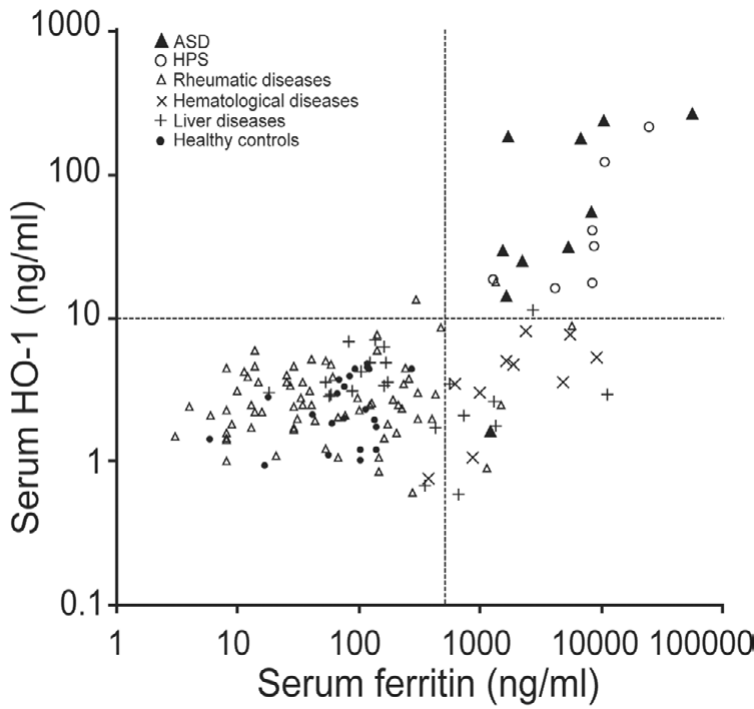
Serum heme oxygenase (HO)-1 and ferritin levels in all the patients studied. Filled triangles stand for patients with active hemophagocytic syndrome (HPS). Open circles stand for those with active adult-onset Still's disease (ASD). The horizontal dotted line indicates 500 ng/ml of ferritin, which was determined on the basis of revised Diagnostic Guidelines for hemophagocytic lymphohistiocytosis [22,25], and the vertical dotted line indicates the arbitrary cutoff value 10 ng/ml of HO-1.

Besides being found in patients with HPS and ASD, hyperferritinemia is also found in patients with liver diseases and in recipients of frequent blood transfusions. Because ferritin synthesis is stimulated by Fe^2+^, which is generated by HO-1-mediated heme degradation, hyperferritinemia might be caused by high HO-1 activity, irrespective of the underlying diseases. To examine this possibility, the relation between serum HO-1 and ferritin was evaluated in all patient groups. A total of 37 patients had serum ferritin levels >500 ng/ml, which is the cutoff level in the revised diagnostic criteria for HLH [22,25]. Serum HO-1 levels exceeded 10 ng/ml in 7 of 7 HPS patients and in 9 of 10 ASD patients but in only 2 of 20 patients with other diseases (one with dermatomyositis and the other with Epstein–Barr hepatitis) (Fig. 4). Of all the subjects studied, only one person, with dermatomyositis, had serum HO-1 >10 ng/ml but serum ferritin <500 ng/ml. Thus, simultaneous elevation of serum ferritin and HO-1 was much more common in patients with ASD and HPS than any other disease studied.

### HO-1 is up-regulated in PBMCs from some, but not all, patients with active HPS and ASD

**Figure 5:**
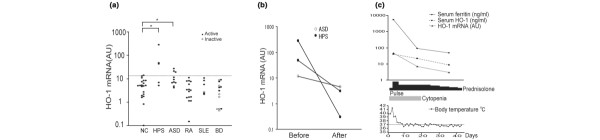
Expression of HO-1 mRNA in PBMCs semiquantified by real-time PCR. The data are expressed as arbitrary units (AU). **(a) **Heme oxygenase (HO)-1 mRNA levels in patients with hemophagocytic syndrome (HPS) (*n *= 5), adult-onset Still's disease (ASD) (*n *= 10), rheumatoid arthritis (RA) (*n *= 15), systemic lupus erythematosus (SLE) (*n *= 6), or Behçet's disease (BD) (*n *= 13), and in normal controls (NC) (*n *= 20). Filled circles and open circles represent patients with active and inactive disease, respectively. *P < 0.05 as determined by the Mann–Whitney *U *test. The horizontal dotted line represents the mean + 2 standard deviations of the mRNA level in healthy controls. **(b) **HO-1 mRNA levels in peripheral blood mononuclear cells (PBMCs) from HPS and ASD patients (filled and open circles, respectively) before and after remission. **(c) **Clinical course of ASD and HPS in one patient. The numbers on the vertical axis representng/ml (serum ferritin and HO-1 concentrations) and AU (HO-1 mRNA). 'Pulse' represents intravenous infusion of methylprednisolone at 1,000 mg/day for 3 days.

**Figure 6:**
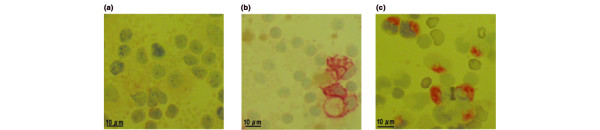
Expression of heme oxygenase (HO)-1 protein in PBMCs, determined using anti-HO-1 monoclonal antibody. **(a) **Untreated peripheral blood mononuclear cells (PBMCs) from a healthy control. **(b) **100 μM hemin-treated PBMCs from a healthy control. **(c) **PBMCs from a patient with active adult-onset Still's disease (ASD) complicated by hemophagocytic syndrome (HPS). HO-1-expressing monocytes (stained red) were found in (b) and (c). Original magnification × 400.

Yachie and colleagues reported that PBMCs from children with acute inflammatory illness express elevated HO-1 mRNA levels [37]. In our study, HO-1 mRNA expression in PBMCs was semiquantified using real-time PCR. We found that PBMCs from 3 of 5 patients with active HPS and 3 of 10 with active ASD had HO-1 mRNA expression exceeding the mean + 2 standard deviations of healthy controls, whereas no such elevations were found in PBMCs from patients with other rheumatic diseases, irrespective of disease activity (Fig. 5a). The six patients with increased mRNA expression universally manifested elevated serum HO-1 protein levels. Moreover, HO-1 mRNA expression fell when remission was induced in two patients with HPS and one with ASD (Fig. 5b). Changes in HO-1 mRNA expression mirrored changes in serum HO-1 protein levels in a 45-year-old woman (Fig. 5c). This patient, who had had ASD for 4 years and maintained remission with PSL (20 mg/day), was admitted to our hospital because of high fever and cytopenia. Bone marrow aspiration revealed hemophagocytosis, indicating that the patient's ASD was complicated with HPS. Besides increased serum ferritin (8,690 ng/ml) and HO-1 (40.4 ng/ml), HO-1 mRNA expression in PBMCs was much higher than that of healthy controls. Immunocytochemistry showed that HO-1-expressing cells were found in hemin-treated, but not untreated, PBMCs from normal donors (Fig. 6a,b), whereas HO-1 proteins were stained in freshly isolated PBMCs, mainly monocytes, from the patient (Fig. 6c). After clinical remission was achieved by mPSL pulse therapy and subsequent oral PSL, HO-1 mRNA in PBMCs was reduced in parallel with serum HO-1 and ferritin levels (Fig. 5c). These data indicate that circulating PBMCs may contribute to increased serum HO-1 protein levels in some subjects. However, since HO-1 mRNA expression was normal in PBMCs from 9 of 15 patients with active HPS and ASD, despite elevated serum HO-1, it is clear that PBMCs are not a critical source of circulating HO-1.

## Discussion

This study demonstrates that serum HO-1 levels are elevated in patients with active HPS and ASD, and that these levels correlate closely with disease activity, irrespective of underlying conditions and clinical phenotypes. Serum HO-1 levels were also slightly elevated in some patients with DM/PM, but not to the degree of patients with HPS or ASD.

Yachie and colleagues reported that HO-1 mRNA levels were elevated in PBMCs from children with acute inflammatory illness and suggested that HO-1 is up-regulated when cells are stressed [37]. It has been shown that HO-1 is cytoprotective in a number of pathological conditions [1,2], although an excess of HO-1 can also injure cells [38-40]. In the current study, increased serum HO-1 was present only in patients with active disease, although it is unclear whether HO-1 was playing a protective or harmful role in these subjects.

Very high levels of serum ferritin are widely used as a marker for HPS and ASD [20,21,23], although the mechanism underlying this increase in ferritin is unknown. The current work documents a significant correlation between serum HO-1 and ferritin levels in HPS and ASD patients. Increased HO-1 activity generates Fe^2+^, a heme catabolyzed product of HO-1, which acts as a potent stimulator of ferritin synthesis [19]. Indeed, it has been shown that more Fe^2+ ^is sequestered by ferritin in ASD patients than in healthy controls, whereas the iron saturation of individual ferritin molecules was decreased [41]. These findings are compatible with the hypothesis that increased HO-1 contributes to hyperferritinemia in ASD and HPS. Alternatively, because Nrf2 (nuclear factor, erythroid derived 2, like 2) regulates transcription of HO-1 and ferritin genes, activation of the transcription factor may be involved in simultaneous overproduction of both molecules [42,43]. On the other hand, it is plausible that an HO-1-independent or an Nrf2-independent mechanism or both are responsible for the elevation in serum ferritin level in subjects with liver disease and frequent transfusions.

Sources of circulating HO-1 in patients with HPS and ASD remain undetermined. These diseases are recognized as macrophage-activation diseases, because increased proinflammatory cytokines such as IL-6, TNF-α, and IL-18 are dominantly produced by macrophages [20,21,25,35,36]. Moreover, HPS and severe ASD are characterized by the proliferation of macrophages that phagocytose hematopoietic cells in the bone marrow and their subsequent infiltration into other organs, accounting in part for the systemic clinical symptoms of these diseases [20]. In response to various stresses, HO-1 is strongly expressed in cells of the macrophage lineage, including circulating monocytes [37]. We found that PBMCs from some, but not all, HPS and ASD patients with elevated serum HO-1 levels overexpressed HO-1 mRNA. It therefore seems that serum HO-1 proteins may be partly derived from circulating monocytes in ASD and HPS patients, although other sources of HO-1 must also be involved.

Useful diagnostic criteria for familial hemophagocytic lymphohistiocytosis [22,25] are well established, whereas it is sometimes hard to diagnose secondary HPS, especially in adults. Although the diagnosis requires the histological identification of hemophagocytosis in organs, the findings are often difficult to prove even by biopsies of the bone marrow, lymph nodes, and liver [21,44]. Depressed NK cell activity and increased soluble IL-2 receptor levels are helpful but are not specific for the disease. In the early stage of ASD, the diagnostic criteria [26,27] are not satisfied in some patients.

Hyperferritinemia is found not only in HPS and ASD, but also in other rheumatic diseases, liver diseases, and hematological disorders with frequent transfusions. All of these diseases can be accompanied by cytopenia and/or high fever, leading to difficulty of differential diagnosis. Since no disease-specific findings have been established, it is important to exclude other diseases. The delay associated with examinations may delay the initiation of critically needed therapies. On the other hand, it is prompt, simple, noninvasive, and informative to measure serum HO-1 levels by ELISA in such situations.

In contrast to the case with HPS and ASD, hyperferritinemia is not associated with elevated serum HO-1 levels in patients with liver disease or hematological diseases requiring frequent transfusions. This clear distinction suggests that the combination of increased serum HO-1 plus ferritin provides greater specificity in the diagnosis of HPS and ASD.

## Conclusion

The present study shows that serum HO-1 is a novel marker for the diagnosis of HPS and ASD and for monitoring disease activity. Further studies are required to determine the mechanism and sources of increased serum HO-1 in these diseases. Clarification of the relation between HO-1 and ferritin metabolism will shed further light on the pathogenesis of HPS and ASD.

## Abbreviations

ASD = adult-onset Still's disease; BD = Behçet's disease; CO = carbon monoxide; CRP = C-reactive protein; DM/PM = dermatomyositis/polymyositis; ELISA = enzyme-linked immunosorbent assay; GAPDH = glyceraldehyde-3-phosphate dehydrogenase; HO = heme oxygenase; HPS = hemophagocytic syndrome; IL = interleukin; mPSL = methylprednisolone; NK = natural killer; PBMC = peripheral blood mononuclear cell; PCR = polymerase chain reaction; PSL = prednisolone; RA = rheumatoid arthritis; SLE = systemic lupus erythematosus; TNF = tumor necrosis factor.

## Competing interests

The authors have received no financial support or other benefits from commercial sources for the work reported in the manuscript, and no other financial interests that any of the authors may have could create a potential conflict of interest or the appearance of a conflict of interest with regard to the work.

## Authors' contributions

YI designed and organized the study. YK, MT, and MI, conducted the laboratory work. YK, MT, AU, SO, AS, HK, KT, and YI were involved in the analysis and interpretation of data. YK, MT, and YI were involved in writing the report. All authors read and approved the final manuscript.

## Figures and Tables

**Table 1:** Characteristics of the patients enrolled in the study

Diagnosis	No.	Age	Sex (M/F)	Serum HO-1 (ng/ml)	Serum ferritin (ng/ml)
***Hemophagocytic syndrome***	7	42.7 (15.5)	1/6	71.2 (72.7)	8485.3 (8388.0)
***Adult-onset Still's disease***	10	41.0 (11.9)	5/5	102.8 (102.6)	9658.5 (17042.1)
***Rheumatic diseases***	73	48.2 (15.9)	22/51	3.4 (2.7)	225.5 (709.9)
Rheumatoid arthritis	30	53.1 (13.2)	8/22	2.8 (1.4)	83.0 (95.3)
Systemic lupus erythematosus	18	38.0 (16.8)	1/17	3.2 (1.9)	188.0 (286.3)
Active	12	42.5 (16.4)	1/11	3.6 (2.1)	220.4 (329.8)
Inactive	6	29.0 (15.1)	0/6	2.2 (1.0)	100.6 (122.1)
Behçet's disease	16	47.3 (14.4)	10/6	2.4 (0.8)	48.3 (43.0)
Active	6	46.5 (14.3)	5/1	2.5 (0.5)	61.4 (45.8)
Inactive	10	47.7 (15.3)	5/5	2.3 (1.0)	40.4 (41.7)
Dermatomyositis/polymyositis	9	54.4 (16.6)	3/6	7.4 (5.2)	1097.2 (1827.9)
***Liver diseases***	20	47.8 (18.1)	16/4	3.7 (2.4)	1032.2 (2496.9)
Acute hepatitis	5	27.8 (5.6)	4/1	4.8 (3.9)	1347.0 (861.0)
Chronic hepatitis	7	51.9 (12.5)	6/1	3.8 (1.6)	159.9 (128.0)
Liver cirrhosis	2	69.0 (7.1)	2/0	5.8 (1.4)	102.0 (28.3)
Hepatocellular carcinoma	2	59.5 (10.6)	2/0	1.9 (0.7)	260.0 (134.4)
Primary biliary cirrhosis	2	46.0 (21.2)	1/1	2.8 (3.0)	373.5 (892.4)
Autoimmune hepatitis	1	34	0/1	2.9	11262
Alcoholic hepatitis	1	71	1/0	2.8	56
***Hematological diseases***	10	62.3 (15.3)	6/4	4.3 (2.4)	2822.6 (2817.3)
Myelodysplastic syndrome	6	59.3 (22.5)	4/2	3.7 (2.6)	1883.8 (1885.9)
Aplastic anemia	4	54.0 (18.8)	2/2	5.1 (2.1)	4230.8 (3671.1)
***Healthy controls***	22	30.8 (7.6)	16/6	2.6 (1.3)	93.0 (56.9)

Data are shown as means (standard deviations). F, female; HO, heme oxygenase; M, male.
